# Adopting transfer learning for neuroimaging: a comparative analysis with a custom 3D convolution neural network model

**DOI:** 10.1186/s12911-022-02054-7

**Published:** 2022-12-07

**Authors:** Amira Soliman, Jose R. Chang, Kobra Etminani, Stefan Byttner, Anette Davidsson, Begoña Martínez-Sanchis, Valle Camacho, Matteo Bauckneht, Roxana Stegeran, Marcus Ressner, Marc Agudelo-Cifuentes, Andrea Chincarini, Matthias Brendel, Axel Rominger, Rose Bruffaerts, Rik Vandenberghe, Milica G. Kramberger, Maja Trost, Nicolas Nicastro, Giovanni B. Frisoni, Afina W. Lemstra, Bart N. M. van Berckel, Andrea Pilotto, Alessandro Padovani, Silvia Morbelli, Dag Aarsland, Flavio Nobili, Valentina Garibotto, the Alzheimer’s Disease Neuroimaging Initiative, Miguel Ochoa-Figueroa

**Affiliations:** 1 Center for Applied Intelligent Systems Research (CAISR), Halmstad University, Halmstad, Sweden; 2 National Cheng Kung University in Tainan, Taipei City, Taiwan; 3 Department of Clinical Physiology, Institution of Medicine and Health Sciences, Linköping, Sweden; 4 Department of Nuclear Medicine, Medical Imaging Area, La Fe University Hospital, Valencia, Spain; 5 Servicio de Medicina Nuclear, Hospital de la Santa Creu i Sant Pau, Universitat Autónoma de Barcelona, Barcelona, Spain; 6 Nuclear Medicine Unit, IRCCS Ospedale Policlinico San Martino, Genoa, Italy; 7 Department of Diagnostic Radiology, Linköping University Hospital, Linköping, Sweden; 8 Department of Medical Physics, Linköping University Hospital, Linköping, Sweden; 9 National Institute of Nuclear Physics (INFN), Genoa section, Genoa, Italy; 10 Department of Nuclear Medicine, University Hospital, LMU Munich, Munich, Germany; 11 Department of Nuclear Medicine, Inselspital, University Hospital Bern, Bern, Switzerland; 12 Laboratory for Cognitive Neurology, Department of Neurosciences, KU Leuven, Belgium; 13 Neurology Department, University Hospitals Leuven, Leuven, Belgium; 14 Department of Neurology, University Medical Centre, Ljubljana, Slovenia; 15 Faculty of Medicine, University of Ljubljana, Ljubljana, Slovenia; 16 Department of Clinical Neurosciences, Geneva University Hospitals, Geneva, Switzerland; 17 LANVIE (Laboratoire de Neuroimagerie du Vieillissement), Department of Psychiatry, University Hospitals, Geneva, Switzerland; 18 VU Medical Center Alzheimer Center, Amsterdam, The Netherlands; 19 Department of Radiology and Nuclear Medicine, Amsterdam Neuroscience , Amsterdam UMC, Vrije Universiteit Amsterdam, Amsterdam, The Netherlands; 20 Neurology Unit, Department of Clinical and Experimental Sciences, University of Brescia, Brescia, Italy; 21 Nuclear Medicine Unit, IRCCS Ospedale Policlinico San Martino, Genoa, Italy; 22 Centre for Age-Related Medicine (SESAM), Stavanger University Hospital, Stavanger, Norway; 23 Department of Neuroscience (DINOGMI), University of Genoa, Genoa, Italy; 24 Division of Nuclear Medicine and Molecular Imaging, University Hospitals and NIMTLab, Geneva University, Geneva, Switzerland; 25 Center for Medical Image Science and Visualization (CMIV), Linköping University, Linköping, Sweden; 26 Department of Biomedical Sciences, University of Antwerp, Antwerp, Belgium; 27 Department of Old Age Psychiatry, Institute of Psychiatry, Psychology and Neuroscience, King’s College London, London, England

**Keywords:** Convolution Neural Networks, Transfer Learning, Brain Neurodegenerative Disorders, Medical Image Classification

## Abstract

**Background:**

In recent years, neuroimaging with deep learning (DL) algorithms have made remarkable advances in the diagnosis of neurodegenerative disorders. However, applying DL in different medical domains is usually challenged by lack of labeled data. To address this challenge, transfer learning (TL) has been applied to use state-of-the-art convolution neural networks pre-trained on natural images. Yet, there are differences in characteristics between medical and natural images, also image classification and targeted medical diagnosis tasks. The purpose of this study is to investigate the performance of specialized and TL in the classification of neurodegenerative disorders using 3D volumes of 18F-FDG-PET brain scans.

**Results:**

Results show that TL models are suboptimal for classification of neurodegenerative disorders, especially when the objective is to separate more than two disorders. Additionally, specialized CNN model provides better interpretations of predicted diagnosis.

**Conclusions:**

TL can indeed lead to superior performance on binary classification in timely and data efficient manner, yet for detecting more than a single disorder, TL models do not perform well. Additionally, custom 3D model performs comparably to TL models for binary classification, and interestingly perform better for diagnosis of multiple disorders. The results confirm the superiority of the custom 3D-CNN in providing better explainable model compared to TL adopted ones.

## Background

Neurodegenerative disorders have a huge negative impact on the healthcare systems globally. Alzheimer’s Disease (AD) is highly prevalent in the elder population, is considered to be the most common disorder with approximately 60% of all dementia [1]. Dementia with Lewy bodies (DLB) is second most common neurodegenerative disorder, increasing prevalence estimates were reported with increasing age. DLB accounted for from 0.3 to 24.4% of all cases of dementia in the prevalence studies [2]. Mild cognitive impairment (MCI) is a transition stage between normal aging and dementia and 10-15% of patients diagnosed with MCI progress every year to dementia, most commonly AD [3].

Different neuroimaging techniques such as magnetic resonance imaging (MRI), positron emission tomography (PET) and single-photon emission computed tomography (SPECT) are proficient to document the functional and anatomical abnormalities informative to diagnose the type of the neurodegeneration [4]. F-18 fluorodeoxyglucose positron emission tomography (18F-FDG-PET) scans which measures cerebral glucose metabolism, has been reported as an accurate biomarker for the discrimination of the above-mentioned neurodegenerative disorders [5].

Different deep convolutional neural network (CNN) techniques have proven to be effective in supporting the diagnosis of most common types of dementia such as AD, MCI, and DLB using 18F-FDG-PET brain images. These techniques show ability to extract features and identify disease-related patterns in imaging input data without prior-knowledge about the pathophysiological mechanisms of the underlying diseases [6–9]. However, one of the main challenges with analyzing medical imaging is that data is limited and expensive to collect. Therefore, transfer learning (TL) becomes a key component of many successful models used for medical diagnosis [7, 9]. The core of TL is to use the knowledge of pre-trained models on a source dataset, and fine-tune it for a target task on a different but related dataset [10, 11].

There are many CNNs well-trained on ImageNet with differing accuracy. These networks have been trained to recognize objects from a huge natural-image dataset which consists of 14 million images of roughly 1,000 different categories [12, 13]. The application of these pre-trained models to neuroimaging studies is an active research field for their expectations on improving classification performance. Specifically, TL models have shown to be timely efficient in classifying AD dementia patients and achieving a good performance [7, 14, 15].

**Fig. 1:**
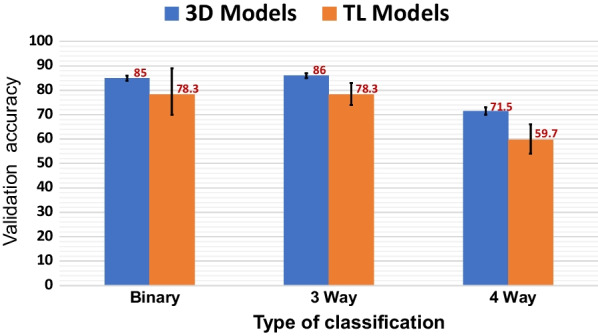
The average validation accuracy obtained for different classification tasks using transfer learning (TL) models and custom 3D models. The error bars show minimum and maximum values obtained during training of different models. Detailed comparison can be found at Table  4

Despite the popularity of TL models in medical imaging, there has been little work studying its precise effects in the medical imaging settings. Particularly, medical tasks often have significantly fewer classes than the standard ImageNet classification. Additionally, TL models are pre-trained with 2D images and thus spatial information is lost during the transformation of the 3D medical images into the 2D space. Furthermore, the nature of features used as representation generated for 3D neuroimaging volumes using pre-trained TL models need further investigation especially with the high resolution non-RGB nature of input data and the low contrast between brain regions and background of scans after data normalization.

Given these open issues with lack of scientific studies highlighting the differences among adopting TL models and training specialized models for medical imaging, in this paper the aim is to compare the performance of deep and transfer learning applied to neuroimaging volumes for classification purposes. The goal is to study the performance of these techniques with respect to multiple aspects. The first dimension in our empirical analysis represents the type of classification task, such as binary classification when distinguishing AD from Cognitively Normal (CN) cases, 3-way classification by adding DLB cases, and finally 4-way classification by considering MCI cases. Additionally, we compare performance of these models in terms of training overhead, obtained accuracy, explainability, and sensitivity towards other similar types of dementia.

In this paper, we use InceptionV3 [16], VGG16 [17], and ResNet50 [18] pre-trained TL models using ImageNet. Furthermore, we develop a 3D CNN that adopts VGG16 in its architecture, yet with less number of convolution blocks [19]. We analyze the classification performance of TL models for detecting multiple Neurodegenerative disorders. Additionally, we discuss the challenges in developing a custom 3D model using limited data, specifically choosing the CNN architecture and addressing overfitting that leads to poor model performance. Figure 1 highlights one of our main perspectives regarding the comparison between adopting TL models and training a custom 3D model from scratch. As shown, both TL models and custom models achieve almost similar performance in binary classification (AD versus CN), however, differences in performances appear when the objective changes to diagnose more disorders. The contributions of this study can be listed as the following:

1. We evaluate the performance of TL models trained with natural images such as ImageNet, as well as custom models for diagnosis of brain disorders using 3D medical imaging data for different classification tasks.
2. We investigate whether using pre-trained TL models lead to different learned representations, by visualizing the generated feature representation by different models. More importantly, we analyze network attention to demonstrate the significant areas of interest indicated by the adopted and developed models. Furthermore, we evaluate robustness and sensitivity of different models towards other similar dementia types.

The rest of the paper is organized as follows: in Section Results we present obtained performance from TL and 3D custom model in different experiments, then we provide discussion and related work, lastly we conclude our paper. Section Methods presents the design of comparative analysis performed between the different models.

## Results

We start by discussing the dataset and the choice of hyperparameters for the developed 3D models. Then, the results of performance comparison among different models are presented. Lastly, we detail our experiments on network attention for explaining the decisions taken by different models.

All of the experiments were conducted using Tensorflow and Keras libraries on a computer with Linux Ubuntu 18.09 operating system, that has one Nvidia Quadro GV100 GPU card with 32GB of memory and 36 CPU core Xenon with 128 GB of memory.

### Neuroimaging dataset

Our primary dataset was collected retrospectively from two different sources as detailed in Table 1. The anonymized scans from patients with probable DLB were collected from the European DLB (EDLB) Consortium [20] having the local institutional ethics committee approvals including the transfer of anonymized imaging brain 18F-FDG-PET scans. Recruited patients were referred to and assessed at outpatient clinics including memory, movement disorders, geriatric medicine, psychiatric, and neurology clinics as previously described in [21]. The diagnosis of probable DLB was originally made according to diagnostic criteria for probable DLB as defined by [22].

**Table 1:** The demographics of the used dataset collected from ADNI and EDLB sites, showing the average age in men and women per each clinical diagnosis (class), and percentage of samples used for training, validation and testing

		Cases (%)	Average age	
Class	Cases	women	Men	Women
Source: ADNI				
AD	200	72 (36.0%)	76.7 ± 8.2	74.0 ± 7.8
MCI	200	76 (38.0%)	75.6 ± 7.5	73.2 ± 8.2
CN	156	62 (39.7%)	77.5 ± 5.4	78.3 ± 5
Source: EDLB				
DLB	157	59 (37.5%)	73.3 ± 7.2	74.8 ± 6.4
CN	44	22 (50.0%)	70.1 ± 10.3	67.5 ± 9.2
Total	757	291 (38.4%)	75.5 ± 7.6	74.4 ± 7.7

The EDLB also provided some normal cases that we added to the CN cases. In total EDLB provided 201 scans from 2005 to 2018. The rest of the images, i.e. 556 scans, were collected from the Alzheimer’s Disease Neuroimaging Initiative (ADNI) [23] across ADNI-1, ADNI-2, ADNI-3 and ADNI-GO (Grand Opportunities) studies from December 2005 to March 2020 [24]. We also used eight Frontotemporal lobar degeneration (FTLD) 18F-FDG-PET scans that were downloaded from the Frontotemporal Lobar Degeneration Neuroimaging Initiative (FTLDNI) database.

Table 1 summarises the demographics of the data collected for this study from EDLB and ADNI. The dataset consisted of 757 cases including 200 AD (from ADNI), 200 MCI (from ADNI), 157 DLB (from EDLB), and 200 CN (156 cases from ADNI and 44 cases from EDLB). We split the data into two sets with 89% and 11% for training and testing using stratified random sampling to keep enough cases from all four disorders while considering the two sources (in specific CN which contains cases both from ADNI and EDLB).

### Classification algorithms

We used InceptionV3, VGG16,and ResNet50 models to be evaluated as transfer learning approach which being trained with ImageNet. Furthermore, we trained a 3D VGG model from scratch using our 18F-FDG-PET scans. Table 2 lists the details of used models with respect to number of trainable parameters. In the following subsections, we describe the different pipelines adopted for performing the classification tasks using TL and custom 3D models.

**Table 2:** Specification of used models in terms of number of trainable parameters, size of generated features and reported accuracy on ImageNet for TL models

Model	Parameters	Features	Accuracy on ImageNet [25]
InceptionV3	23,851,784	2,048	94.49%
VGG16	138,357,544	512	91.9%
ResNet50	25,636,712	18 x 25 x 2048	92.9%
3D Model	62,997,012	1,024	-

### 3D CNN model specification

VGG16 was designed for 2D images with small and fixed filters across all the convolution layers (i.e. filters of size $3 \times 3$). In order to add depth (i.e. 3D), one choice could be keeping a homogeneous filter of size $3 \times 3 \times 3$ across all convolution layers. However, we wanted to investigate the performance using different depth values, e.g., having a filter $3 \times 3 \times 6$. Also, as we are handling 3D data, we wanted to study the effect of treating each slice from the input 18F-FDG-PET scan separately in the first convolution layer. Therefore, we developed four models, each with a different structure in terms of kernel shape across the convolution layers. We performed the experiments with end-to-end training using mini-batches of size 6 and Adadelta optimizer with 0.01 learning rate for 50 epochs. Additionally, to prevent the model from overfitting we used early stopping condition by monitoring the validation loss in order to end the model training when the model performance stops improving (i.e., less than 0.0001 change in validation loss for 10 epochs).

We evaluated the different models through 10 rounds of K-Fold Cross Validation (KFCV) on the training set, also computed the 95% confidence intervals, the results are shown in Table 3. As results indicate, there is no huge difference among obtained accuracy using different kernels. So, we choose to keep a homogeneous 3x3x3 filter as it achieves the highest training and validation accuracy for the different folds during the cross validation evaluation.

**Table 3:** Performance of 3D model alternatives designed with different values for depth in convolution kernels.

	L(1): Conv(3x3x1) followed by L(r):Conv(3x3x3)		L(a): Conv(3x3x3)		L(1): Conv(3x3x1) followed by L(r): Conv(3x3x6)		L(a): Conv(3x3x6)	
CV	T acc.	V acc.	T acc.	V acc.	T acc.	V acc.	T acc.	V acc.
2-F	0.76 ±0.04	0.58±0.12	**0**.**77** ±0.03	**0**.**59** ±0.14	0.69 ±0.04	0.42 ±0.12	0.7 ±0.03	0.54 ±0.14
3-F	**0**.**83** ±0.04	0.7 ±0.02	**0**.**83** ±0.05	**0**.**71** ±0.03	0.73 ±0.02	0.68 ±0.02	0.73 ±0.02	0.66 ±0.02
4-F	0.79 ±0.02	0.7 ±0.02	**0**.**8** ±0.02	**0**.**7** ±0.02	0.76 ±0.04	0.66 ±0.02	0.75 ±0.04	0.66 ±0.03
5-F	0.8 ±0.04	0.71 ±0.02	**0**.**82** ±0.03	**0**.**72** ±0.02	0.78 ±0.04	0.7 ±0.02	0.75 ±0.03	0.68 ±0.02
6-F	0.82 ±0.03	0.72 ±0.02	**0**.**83** ±0.04	**0**.**72** ±0.02	0.77 ±0.03	0.68 ±0.02	0.76 ±0.03	0.68 ±0.02
7-F	0.81 ±0.03	0.72 ±0.02	**0**.**87** ±0.03	**0**.**72** ±0.02	0.77 ±0.04	0.7 ±0.02	0.77 ±0.03	0.69 ±0.02
8-F	0.84 ±0.03	0.71 ±0.02	**0**.**86** ±0.03	**0**.**72** ±0.02	0.81 ±0.04	0.7 ±0.02	0.76 ±0.03	0.70 ±0.02
9-F	0.86 ±0.03	0.72 ±0.02	**0**.**87** ±0.03	**0**.**72** ±0.02	0.82 ±0.04	0.69 ±0.02	0.82 ±0.04	0.71 ±0.02
10-F	0.86 ±0.03	0.72 ±0.02	**0**.**87** ±0.03	**0**.**72** ±0.02	0.83 ±0.03	0.70 ±0.02	0.82 ±0.03	0.71 ±0.01

L(1) represents the first convolution layer, L(r) represents the remaining convolution layers, L(a) represents all of the convolution layers in the developed 3D model. The values show the obtained accuracy followed by 95% confidence interval, while T and V represent training and validation accuracy, respectively. Bold represents the best value achieved

### Further optimizations

When training a large network, there is a point during training when the model will stop generalizing and start overfitting of the training data. Overfitting results in increasing the generalization error, making the model less useful at making predictions on new data. The challenge is to train the network long enough to make it capable of learning the mapping between inputs and outputs, but not so long to avoid overfitting training data. Particularly, the number of training epochs as a hyperparameter and train the model with an early stopping condition combined with different learning rates and dropout rations. We performed two different experiments to show the effect of different learning strategies (i.e., fixed epoch training vs early stopping), learning rates (e.g., testing different values such as 0.1, 0.01, and 0.001), and dropout (e.g., with values of 0.2, 0.3, 0.4 and 0.5).

**Fig. 2:**
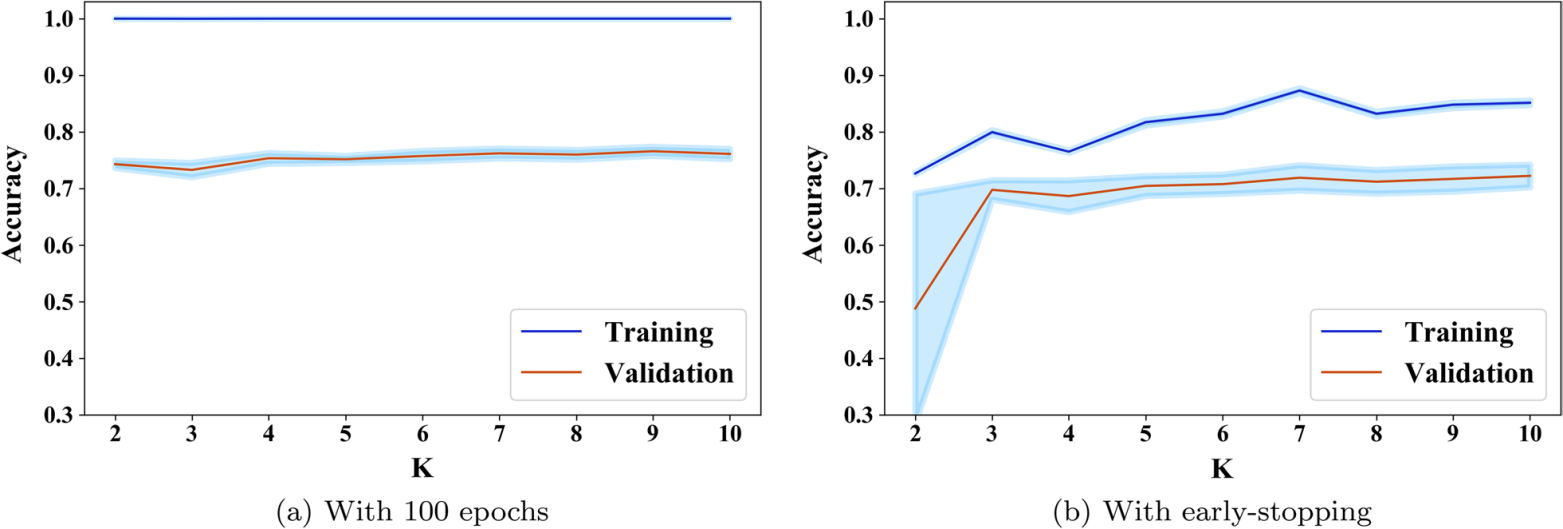
Confidence Intervals (95% CI) for training and validation of the 3D model during KFCV with different stopping criteria. Early stopping condition helps to avoid overfitting training data

In the second experiment, we trained 3D-CNN model for 4-way classification tasks for 10 rounds of K-Fold Cross Validation (CV) on the training set. Also, we computed the 95% confidence intervals. Figure 2 illustrates the obtained results, as shown training strategy with early stopping allows the model to avoid overfitting of training data and provide better generalization for validation data. We use value of 0.0001 as a minimum change in validation loss, and monitor the change for 10 epochs. On the other hand, without early stopping in which we run model training for 100 epochs, the gap between training and validation accuracy is huge. Thus, for the further experiments, we train our 3D custom model using the early stopping condition.

**Fig. 3:**
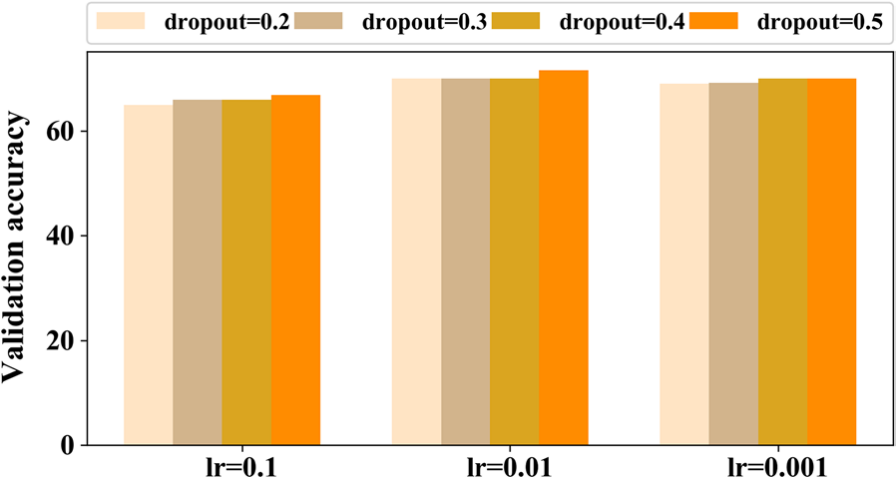
Validation accuracy obtained using 3D custom model with different values of learning rate (lr) and dropout. According to obtained validation accuracy, there is no huge difference while changing lr and dropout

For the third experiment, we trained the model with early stopping training strategy with different values of learning rate (lr) and dropout rations as shown in Fig.  3. According to obtained results, we set lr to 0.01 and dropout to 0.5 as the highest validation accuracy was obtained using these values.

### Performance evaluation on multi-classification tasks

Most DL models applied in neurodegenerative diseases mainly focus on binary classification or classify multiple stages of AD from no dementia to moderate AD. However, the utility of such models is limited to the AD patient population solely, which makes them unable to discriminate non-AD patterns from AD, also it becomes hard to validate their robustness in the presence of non-AD disorders. The proper diagnosis of dementia patients requires going beyond binary classification and at least recognizing the differences among Cognitively Normal (CN), MCI and other dementia types, especially the most common ones such as AD and DLB. Therefore, in the following experiment we evaluate the accuracy of used models using different classification tasks.

In this experiment we evaluated the performance of different models for different classification tasks. We started with binary classification, where the objective is to recognize AD from CN cases. Then, for 3-way classification we added the DLB cases, and lastly 4-way classification is performed to distinguish among the four classes existing in our dataset. In order to perform this experiment, we randomly divided the training data into 80% used for training and 20% used for validation.

For 3D model, we trained our model from scratch using mini-batches of size 6 and Adadelta optimizer with 0.01 learning rate for 50 epochs and Dropout layers with 0.5 rate. Additionally, to prevent the model from overfitting we used early stopping condition as discussed in Experiment 2.

For TL models, we trained a separate classifier for each classification task using the features of cases belonging to training set, then computed the validation accuracy with features representing validation set. We repeated this process for 10 rounds and obtained results are listed in Table 4. We reported the average time in seconds and we also report the standard deviation of the time taken to train classifier for these 10 training rounds. We report the average value for training and validation accuracy, also we computed 95% confidence intervals.

Additionally, we used normalized mutual information (NMI) index by comparing ground-truth labels with the predicted labels generated by different models. Basically, mutual information index is a non-negative quantity and is upper bounded by the values of entropy of identified classes that can measure the information that predicted and ground-truth labels share. The highest value of NMI can be reached when the predicted labels are exactly the same as the data labels.

**Table 4:** Performance of different models (TL and 3D) across the multiple classification tasks (binary, 3-way, 4-way).

Binary classification (AD vs CN)				
Model	Time (SD)	Training acc. (± CI)	Validation acc. (±CI)	NMI
InceptionV3	2.6 (0.5)	**100**	76 ± 0.016	0.94
VGG16	20.7 (0.3)	90 ± 0.006	70 ± 0.044	0.94
ResNet50	**635 (0.2)**	**100**	**89** ± 0.012	**0**.**97**
3D model	108 (0.4)	97 ± 0.012	86 ± 0.042	0.96

3-Way classification (AD vs CN vs DLB)				
Model	Time (SD)	Training acc. (± CI)	Validation acc. (±CI)	NMI
InceptionV3	28.2 (0.7)	99.8 ± 0.0006	78 ± 0.019	0.61
VGG16	2.5 (0.2)	89 ± 0.005	74 ± 0.025	0.6
ResNet50	**877.3 (0.7)**	**100**	83 ± 0.044	0.79
3D model	161 (0.3)	96 ± 0.01	**87** ±0.01	**0**.**9**

4-Way classification (AD vs CN vs DLB vs MCI)				
Model	Time (SD)	Training acc. (±CI)	Validation acc. (± CI)	NMI
InceptionV3	3.6 (0.6)	97 ± 0.006	59 ± 0.01	0.57
VGG16	3.2 (0.1)	69 ± 0.006	54 ± 0.019	0.56
ResNet50	**1193 (0.8)**	**100**	66 ± 0.008	0.62
3D model	296 (0.5)	85 ± 0.026	**73** ± 0.015	**0**.**82**

Acc stands for accuracy, SD indicated standard deviation, CI is used for confidence interval, and NMI represents Normalized Mutual Information index. Bold represents the best value achieved

As shown in Table 4, TL models achieve good performance for binary classification. ResNet50 has the highest validation accuracy. Also, 3D model performed very well, it is the second best in terms of validation accuracy. It is also worth noticing that fine-tuning ResNet50 for our data took more time than training a specialized 3D model from scratch with a simpler network structure.

For classification tasks that consider multiple disorders at the same time, our 3D model performs better than TL models, due to its ability to extract relevant features from input data that make separation boundary among different classes more evident as explained in the results of the next experiment. This also can be shown with achieving highest validation accuracy for 3-way and 4-way classification. Additionally, the values of NMI obtained by our model are the closest to the values computed using ground-truth labels with a prominent difference from values reached by TL models.

### Analysis of 4-way classification

The difference in performance between specialized 3D model and TL models appears in classifying multiple brain disorders. Therefore, in this section we analyze further the features and classification decisions obtained by different models when the objective is to perform 4-way classification.

#### Visualizing extracted feature

**Fig. 4:**
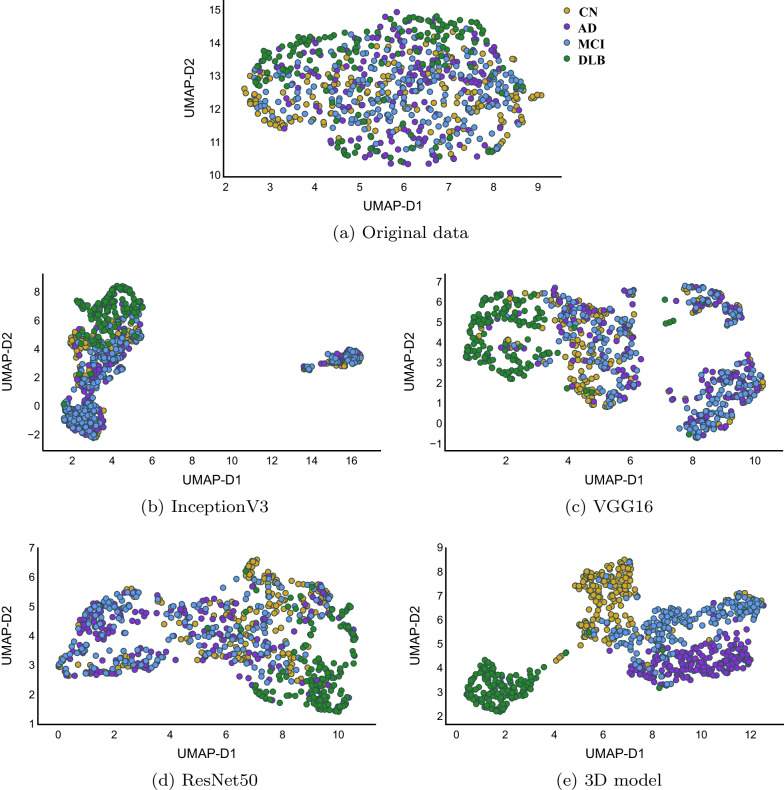
UMAP visualizations for the original data (top), after being passed through the pre-trained models, InceptionV3 **b**, VGG16 **c** , and ResNet50 **d**, finally with 3D custom model **e**. The UMAP embeddings were created using the unsupervised version of UMAP. All subplots maintain the same color scheme for the target classes, i.e., yellow, purple, blue, and green identify CN, AD, MCI, and DLB, respectively using the ground-truth labels

We used the unsupervised UMAP to visualize 1) the original normalized data and 2) the extracted features by different models (before the classification layer) as shown in Fig.  4. The unsupervised UMAP is used to qualitatively evaluate the generated representation by each DL model, specifically, we run UMAP to generate 2D representation of extracted features without specifying any number of classes.

For TL models, some of DLB cases were separable in the representation space, however, rest of cases belonging to CN, AD, and MCI are overlapping that makes it difficult to reach a decision boundary to separate them while training a classifier. On the other hand, features extracted by 3D model made DLB cases very well separated and it is explaining the good performance of the model. The other interesting pattern in Fig.  4 (e) is the distribution of cases from CN to MCI and then to AD, which is as happening in reality: people with CN brains either will develop DLB or they will develop MCI and then AD (if they get dementia, of course).

**Fig. 5:**
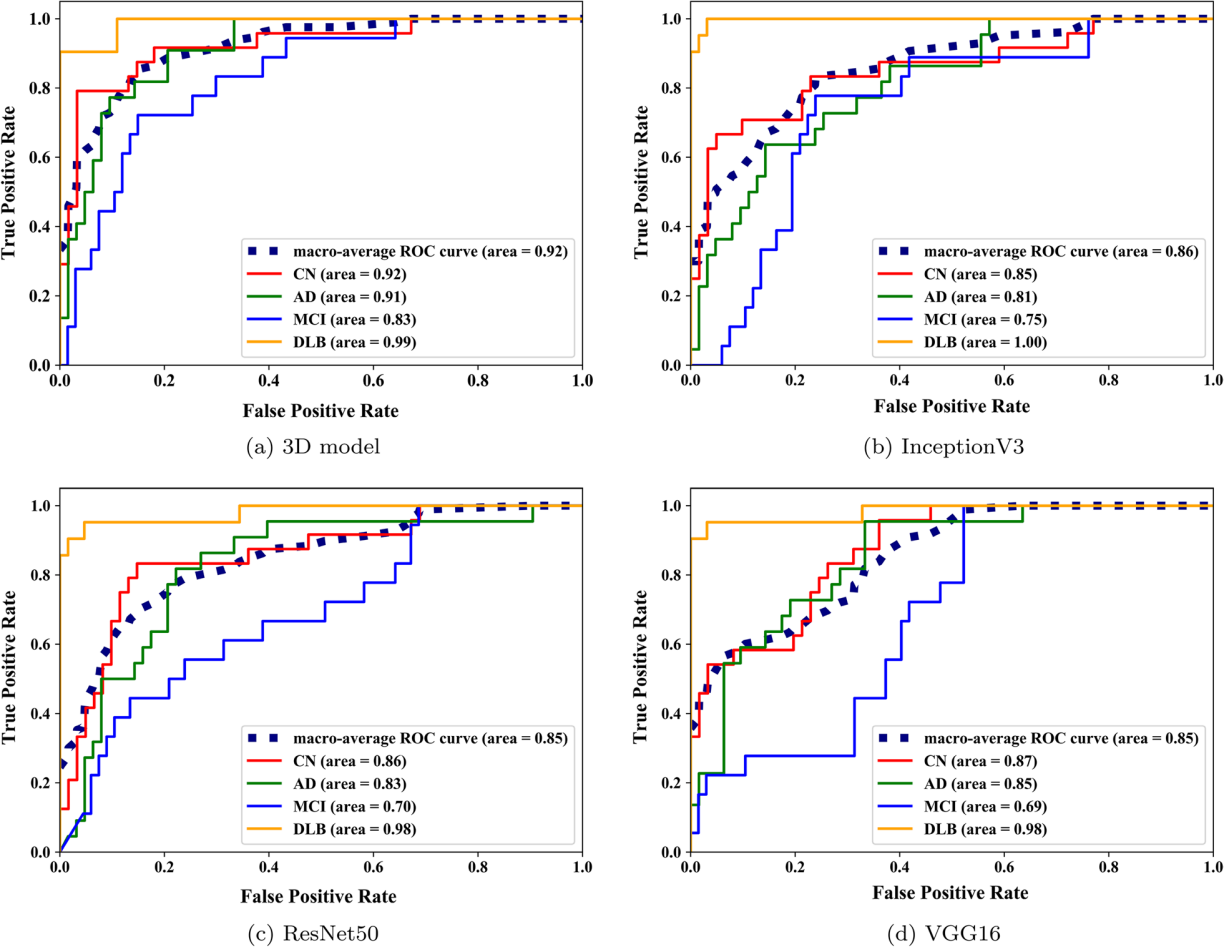
ROC-Curves for 4-way classification for specialized 3D and TL models. 3D model achieves the highest macro-average AUC and performes the best for MCI cases compared to TL models. 3D model has the highest macro-average AUC for all classes, and highest per class types except for DLB

Figure 5 shows receiver operating characteristic (ROC) curves obtained for test set from the different models. As shown, specialized 3D models achieves the highest macro-average with 92% AUC, followed by ResNet50 with 86% AUC, then both InceptionV3 and VGG16 have 85% AUC. It is also interesting to see that all models achieve comparable performance in identifying DLB cases in the test set. However, TL models have the lowest performance in identifying MCI cases.

#### Explainability of model classification

**Fig. 6:**
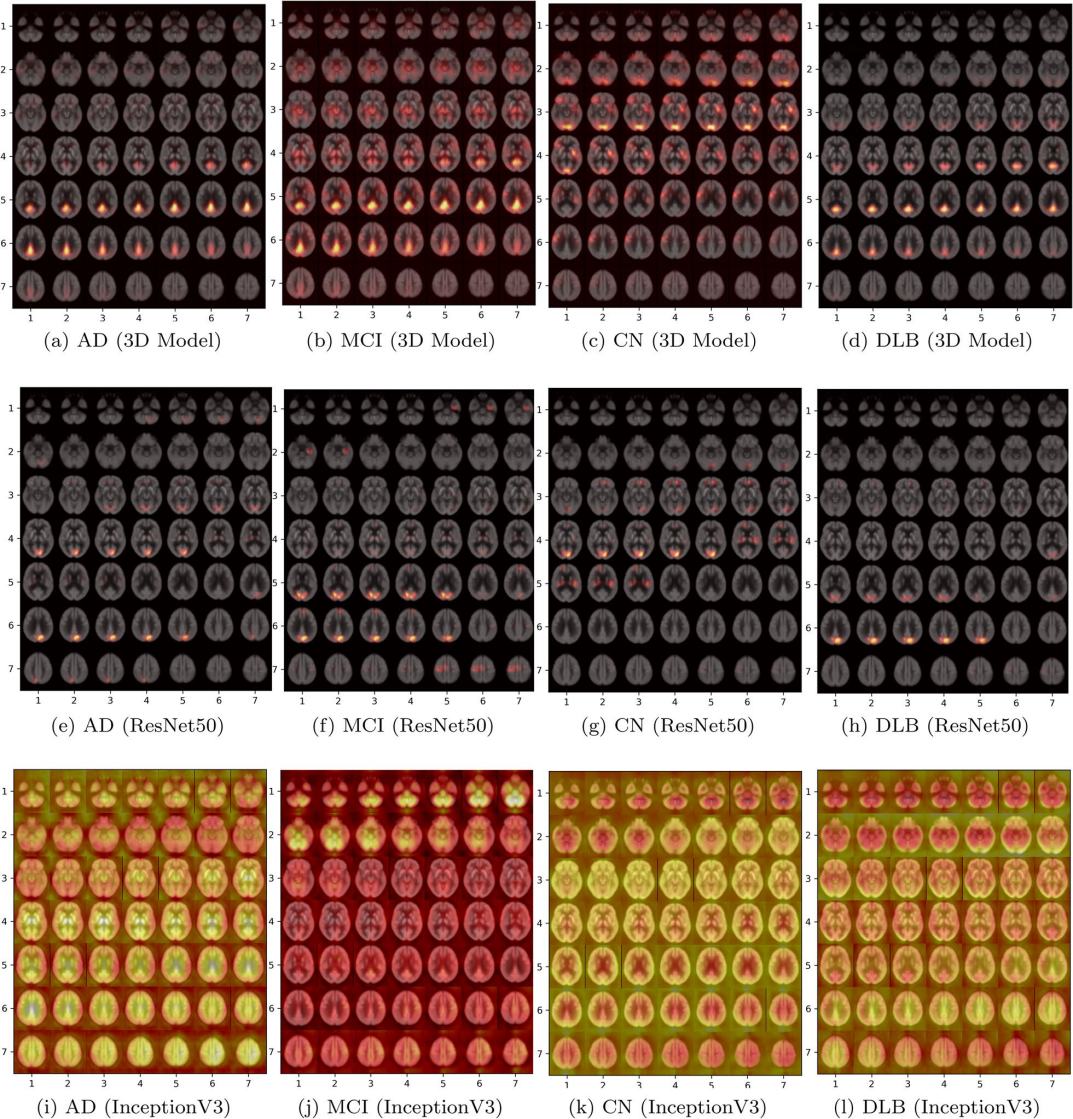
Results of the occlusion experiments for adopted models. The results are projected by creating a mosaic of slices in the axial direction. The cross entropy maps have been over layered with the average brain

Occlusion experiment is used to visualize network towards a specific class. We applied occlusion experiment for ResNet50, InceptionV3 and compare it with results obtained by occlusion on a trained 3D model as explained in Sect. . The results show the cross entropy response of the network given such occluded data as a function of the position of the occlusion box. The experiments were done for all four classes in the training dataset, i.e. when calculating the maps for the DLB class, only DLB subjects were included. The assumption is that when ignoring a relevant region for the correct classification, the cross entropy response will be high.The cross entropy maps are then projected using a mosaic of the slices 5 to 54 (to create a $7 \times 7$ grid) on the axial direction and over layered with the average brain. The occlusion heatmaps visualize metabolism patterns within each class, specifically we show the average cross entropy using all samples belonging to each class separately.

The results are illustrated in Fig.  6. As shown, the highlighted regions in each disorder indicate which brain regions were of more attention from the models in their predictions. Looking at the results, it is clear that the models responded differently to the occluded areas. However, InceptionV3 pays a lot of attention to the background and it is difficult to define discriminative regions that can be linked to each class. On the other hand, ResNet50 shows attention to regions belonging to brain area and these regions differ among classes, however the spatial structure to these regions is lost across the similar consecutive brain slices, e.g., regions highlighted for different classes from third till fourth row. The 3D spatial structure of brain is better maintained with the 3D model showing discriminative regions that are defined for each class type. Thus, we provide clinical explanations for highlighted regions with custom 3D model.

For the 3D model, AD (Fig.  6.a) the posterior cingulate cortex is the most discriminating region among others, while in MCI (Fig. 6.b) pons thalamus and parietal (post-central gyrus/somatosensory cortex) are important in addition to the posterior cingulate cortex. Furthermore, the occipital, left striatum, right frontal cortex, and right parietal (post-central gyrus/somatosensory cortex) are the highlighted regions in CN (Fig. 6.c). And finally in DLB cases (Fig.  6.d) the posterior cingulate cortex is also taking an important role in differentiating DLB besides the occipital cortex.

The posterior cingulate cortex is important for all the given neurodegenerative disorders, i.e., AD, MCI, and DLB, and not in CN. 3D model shows the pattern in this brain region makes the most difference in a cognitively normal brain compared to dementia-involved ones. The other interesting pattern is depicted in MCI and CN maps and probably is the underlying reason for misdiagnosing MCI with CN. The parietal (post-central gyrus/somatosensory cortex) is highlighted in both maps and generally both are sharing many common brain regions of interest.

#### Analysis of model robustness

Our objective of this experiment is to analyze the sensitivity of models towards similar types of dementia to see how the models recognize these cases and create their equivalent representations. Though, the objective of trained models, as any supervised classification task, implies that the input space is projected into a finite set of defined categories (in this case AD, DLB, CN, and MCI). Yet, in this analysis, we investigate the generated representation for FTLD cases and evaluate the labels assigned to them to see if they are grouped together or scattered across the representation space with labels belonging to multiple classes.

Table 5 lists the assigned labels for each one of FTLD cases using 3D and TL models. As shown, specialized 3D model and InceptionV3 assigned only two labels to these cases. For 3D model, half of the cases were recognized as AD, while the rest as CN. InceptionV3 recognized seven out of the eight cases as CN, and the last case was labeled as AD. VGG16 model distributes the FTLD cases among all of the different class types exist in the training data, showing only two as AD. ResNet50 predicted one case as MCI and another one as DLB, while the remaining cases got the AD label.

**Fig. 7:**
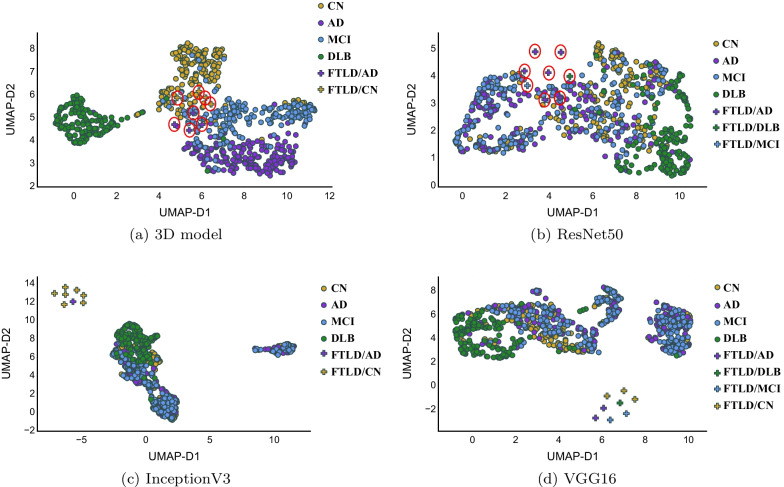
UMAP visualization showing training data and FTLD cases used as external test-set generated by specialized 3D and TL models. In cases of 3D model and ResNet50 the FTLD cases are identified with red circles as they have very close representation to the training data

Figure 7 shows the UMAP visualization of the training data as well as FTLD cases. Interestingly, all models generate representations of FTLD cases in which they are close to each other and not scattered in the representation space. Additionally, specialized 3D model and ResNet50 generate representation for FTLD cases that is close to the training data that could be expected as brain scans cases share many details in general. Classification outcome of InceptionV3 might seem to be most reasonable as majority of cases receive the same label, 7 out of 8 were recognized as CN cases. However, according to previous study that discuss the similarity between AD and FTLD [26], it should be excepted that more cases to be labeled as AD reflecting similar metabolism patterns linked to AD, learnt by the models when being exposed to training data, which is the case with 3D model that recognized 4 of the FTLD cases as AD and ResNet50 which labeled 6 out of 8 cases as AD.

**Table 5:** Predicted labels for FTLD cases using different models

ID	3D Model	ResNet50	InceptionV3	VGG16
FTLD$_{1}$	AD	AD	CN	AD
FTLD$_{2}$	AD	AD	AD	CN
FTLD$_{3}$	AD	AD	CN	CN
FTLD$_{4}$	CN	AD	CN	CN
FTLD$_{5}$	AD	DLB	CN	MCI
FTLD$_{6}$	CN	AD	CN	DLB
FTLD$_{7}$	CN	MCI	CN	MCI
FTLD$_{8}$	CN	AD	CN	AD

## Discussion

Deep CNNs require large amount of data for training in order to achieve good classification performance. However, the medical images are hard to obtain, as the collecting and labeling of medical data confronted with both data privacy concerns and the requirement for time-consuming expert explanations. Clearly, there are significantly more datasets of natural images. Thus, TL models have been fine-tuned and applied in medical domain for various classification tasks using their previous learning from ImageNet dataset.

Medical images typically represent much higher resolution with non-RGB channels volumes. Additionally, classification models for medical applications need to detect patterns that depend on small and local variations in the input data. Due to this huge diversity between natural and medical image modalities, it remains questionable how much ImageNet feature reuse is helpful for medical images? In this study we focus our analysis on neuroimaging, with detecting diagnosis of most common types of dementia (AD, MCI, and DLB) using 18F-FDG-PET scans of the brain.

Designing and training 3D model from scratch can be a challenging task, particularly with limited available data. Too simple models might not be able to learn enough representation of input data, leading to poor performance. On the other hand, training a very complex network with limited data is hard which leads to overfitting. Having a network model with proper size and other effective methods preventing overfitting, such as proper dropout, learning rate, and early stopping, can get the best results. We have demonstrated with different experimentation the effect of these hyperparameters on training 3D model from scratch. Results show that small learning rate leads to a long training process that could get stuck. Furthermore, dropout regularization and early stopping conditions help our shallow 3D model, with only 4 convolutional layers, to avoid overfitting. From a computational-overhead point of view, feature extraction from TL models provides timely efficient solution for binary classification tasks. However, as we show fine-tuning huge pre-trained model (i.e., ResNet50) on target medical data can require huge computational resources and take more time than training a smaller custom 3D model from scratch.

We have demonstrated with three state-of-the-art ImageNet deep architectures and a well-trained 3D model that a customized 3D model can achieve comparable and even better performance for neuroimaging. In our study, we have successfully demonstrated the effectiveness of the different models in distinguishing neurodegenerative brain disorders with binary, 3-way, and 4-way classification tasks. The results show that TL models obtain superior performance in differentiating AD from CN cases, however, performance decreases when adding more disorders to the classification task. Particularly, performance of 3D custom model becomes better when predicting the diagnosis of two or more brain disorders. We have shown the improvements obtained by 3D model for 4-way classification over TL models using AUC, also by visualizing representation space of extracted features.

We have also provided further analysis that goes beyond classification accuracy and demonstrated with occlusion experiments the areas of interest indicated by 3D model, ResNet50, and InceptionV3. The results show that both 3D model and ResNet50 provide heatmaps with specific regions identified per each class. However, TL models were not able to maintain the spatial information among brain regions across the consecutive slices. It is worth mentioning that our results are limited to adopting TL as feature extractors, as we froze the pre-trained weights for convolution layers of TL models and we only fine-tuned fully connected layers to have more specialized classifiers.

### Related work

Pre-training has received much attention in medical image analysis. For example, Nobili et al. [15] introduced a study to compare performance of deep/TL models and support vector machine (SVM) model for the early diagnosis and prognosis of AD using MRI scans by designing different binary classification tasks. They trained a very simple 3D CNN model that has a single convolution layer followed by ReLU activation and max-pooling layer. For SVM model, they adopted a feature selection method to reduce the dimensionality of input. Using this simple structure of custom 3D CNN, the results show that ImageNet pre-trained models outperform SVM and 3D model trained from scratch. Similar results were achieved with a comparative study on a chest X-ray dataset to classify pneumonia [27].

Ding el al [7] used InceptionV3 model for predicting development of Alzheimer’s disease from 18F-FDG-PET scans. The algorithm achieved area under the ROC curve of 0.98. However, interpreting the model decisions using Saliency maps was not successful as the patterns presented were not specific enough to be mapped to human interpretable imaging biomarkers. The occlusion experiment performed in this study showed the same issue with interpreting the decisions of InceptionV3 model.

Few studies shed the light on the limitations of TL in medical imaging. For example, Raghu et al. [28] show that ImageNet pre-training does not improve medical image classification tasks by evaluating the performance of ResNet50 and InceptionV3 models using Retina images for binary classification task and chest x-ray dataset for diagnosing of five different pathologies. Their experiments suggest that the domain mismatch between natural and medical images inhibits transfer learning. The results show that TL models have minimal effect on performance of detecting Diabetic Retinopathy. Also, for chest x-ray dataset, TL models are worse for recognizing Atelectasis, Cardiomegaly, and Consolidation cases. These results are in line with the findings of our work.

Compared to these previous works, our work takes a step forward and studies the effectiveness of adopting TL for different classification tasks. Furthermore, we investigated the generated representation of TL models and compared it with the ones obtained from a custom 3D model. Additionally, we showed using occlusion experiment that the decisions of TL models might not be informative nor related to medical data properties (i.e., the case with InceptionV3).

## Conclusions

To understand the benefits and limits of TL and training specialized models for diagnosis of different brain disorders, we not only look at standard performance metrics, but also include analysis of key properties particularly important to training and fine-tuning models, extracted features representing medical data, and network attentions. The design of TL models is likely to be suboptimal for the classification of neurodegenerative diseases. Specifically, we found that supervised TL can indeed lead to superior performance on diagnosis of AD versus CN in timely and data efficient manner, yet for detecting more than a single disorder, TL models do not significantly help performance. Furthermore, custom 3D trained models perform comparably to TL models for binary classification, and interestingly perform better for diagnosis of multiple disorders. Additionally, the results confirm the superiority of the custom 3D-CNN in providing better explainable model compared to TL adopted ones.

## Methods

### Data preprocessing

The 18F-FDG-PET scans were spatially normalized to match the International Consortium of Brain Mapping (ICBM space template for European brains) template [29]. Subsequently, the probability maps of grey matter, white matter, cerebrospinal fluid, bone and soft tissue/ air were extracted. The skull stripping was done by retaining the voxels with high probability of being grey matter, white matter or cerebrospinal fluid while discarding those likely being bone and soft tissue/air. The normalized and skull stripped scans were then visually inspected to assess their normalization quality and ensure that the spatial normalization converged to an acceptable solution. Both the spatial normalization and skull stripping processes were done using Matlab R2016a and SPM12. All the brains positioned approximately in the center of the volume.

After the spatial normalization step for the input scans, the first 10 slices as well as the last 9 slices of each scan were excluded as they contain very small objects. Thus, we have the input data as a 3D volume of $95 \times 79 \times 60$ for each case from 757 cases in the dataset. Since scans are from various sites, it is required to perform intensity normalization in order to bring voxel intensities to a common scale across the whole dataset. Therefore, we adopted a feature-wise standardization technique provided by Keras library.

**Fig. 8:**
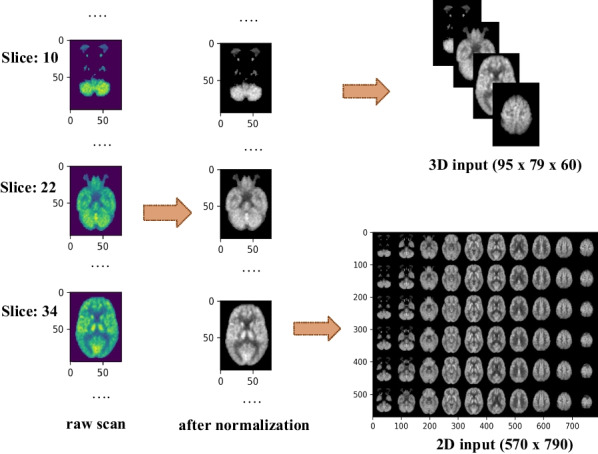
Processing 18F-FDG-PET raw scans for generating 3D as well as 2D inputs for custom 3D model and TL models. For 3D input we build 3D matrix having 60 slices along the axial plane. To transform input for 2D-TL models, we arrange these 60 slices into a 2D grid

The main idea is to treat each 18F-FDG-PET scan separately, then normalize the voxel values using mean and standard deviation. Particularly, we treated each scan as a sequence of 2D images along the axial plane. We applied feature-wise normalization such that each 3D voxel was normalized by subtracting feature-specific mean then dividing by the feature-specific standard deviation per each scan. We performed further scaling to have all intensities values in the range of [0,1]. To transform the input format from 3D to 2D for TL models, we organized the 60 slices of each normalized scan into a 2D grid resulting in having 2D image with $570 \times 790$ pixels for each 18F-FDG-PET scan as shown in Fig. 8. Lastly, input of TL models should have three channels representing RGB color of input images. Our data is considered as gray scale, so we replicate the values across the RGB channels.

### Comparative analysis

**Fig. 9:**
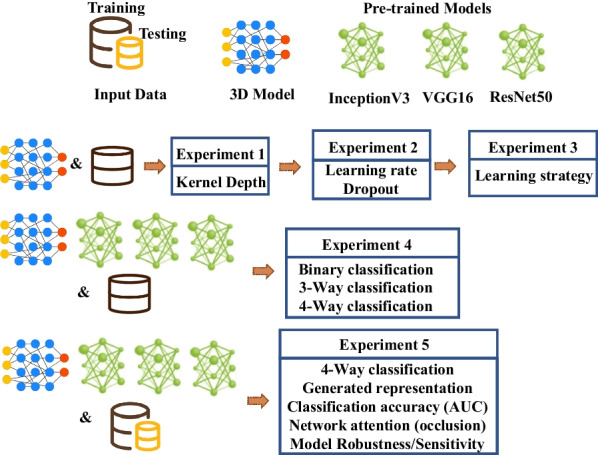
The experiments performed for comparative analysis in this study. We split our data into train and test sets. We have dedicated experiments for tuning custom 3D model as well as experiments for comparative analysis of TL and 3D models

We briefly describe the experiments developed for this study and illustrate the objectives of each experiment. As shown in Fig. 9, the first set of experiments is dedicated to the process of building the CNN from scratch, specifically choosing the hyperparameters to reach an efficient 3D model. Three different experiments were designed to determine kernel depth to be used for the 3D convolution filters, also we tested different learning strategies with and without early stopping conditions to avoid overfitting. Lastly, compared different values for learning rate and dropout.

Additionally, we designed the fourth experiment to compare efficiency of 3D and TL models in discriminating different neurodegenerative disorders with multiple classification tasks. We evaluate the models by performing binary, 3-way, and 4-way classification. Furthermore, our fifth experiment provides detailed comparison of performing 4-way classification. Besides evaluating accuracy of different models, we investigate the learned representations by visualizing the generated feature representation extracted by different models using a dimensionality reduction technique. We used Uniform Manifold Approximation and Projection (UMAP) to produce 2D features of generated representations of input data using adopted models. We chose UMAP as it preserves the global data structure as well as the relative closeness of data points [30].

During the experiments 1 to 4, we adopted 80% and 20% strategy to split data into training and validation datasets. Regarding experiment 5, as we want to report accuracy using AUC under ROC curves, we needed to use the holdout testing dataset for this purpose, thus we split the data into 90% to be used for training and validation and the remaining 10% is used as an independent testset.

We used occlusion to analyze network attention towards significant areas of interest indicated by the models. Occlusion Sensitivity helps to determine whether the output of the model is based on the correct identification of objects with the high sensitivity associated to specific local structures in input images [31]. The occlusion experiment is performed by repeatedly occluding specific regions in the input image and observe the change in the output probability of the model. When important regions for correct classifications are occluded, the probability drops, hence we can observe a significant change in activations of the corresponding feature maps. We performed occlusion sensitively for 3D and TL models using 2D window of size $6 \times 5$ to be removed from each input slice with a stride of $2$. We used 2D window for occlusion to hide the same number of pixels for TL and 3D models.

Our last comparative analysis task is dedicated to evaluate the robustness of the models using brain scans of a new dementia type that was not included in training set. For such sensitivity analysis we used eight Frontotemporal lobar degeneration (FTLD) 18F-FDG-PET scans as another external test set for different models. 18F-FDG-PET brain scan of an FTLD patient is expected to have low FDG uptake in the frontal and temporal lobes [26]. A patient with a chronic AD can eventually have involvement of the frontal lobes and eventually see like a FTLD. Thus, we preformed the last experiment to evaluate the predicted labels of these eight cases using different models, also to visualize the generated representation of these cases and analyze sensitivity of the models towards similar common types of dementia.

## Data Availability

art of data was collected from Alzheimer’s Disease Neuroimaging Initiative (ADNI) at https://adni.loni.usc.edu/.

## Abbreviations

18-F-FDG-PET: 18 F Fluorodeoxyglucose (18F-FDG) Positron emission tomography
2D: Two dimensional
3D: Three dimensional
AD: Alzheimer’s disease
ADNI: Alzheimer’s disease neuroimaging initiative
AUC: Area Under the ROC curve
CI: Confidence Interval
CN: Cognitively normal brain
CNN: Convolution neural network
DL: Deep learning
DLB: Dementia with Lewy bodies
EDLB: European DLB consortium
FTLD: Frontotemporal lobar degeneration
KFCV: K-Fold cross validation
MCI: Mild cognitive impairment
NMI: Normalized mutual information
ROC: Receiver operating characteristic curve
TL: Transfer learning
UMAP: Uniform manifold approximation and projection
